# Transcript profiling of the immunological interactions between *Actinobacillus pleuropneumoniae* serotype 7 and the host by dual RNA-seq

**DOI:** 10.1186/s12866-017-1105-4

**Published:** 2017-09-12

**Authors:** Ping Li, Zhiwen Xu, Xiangang Sun, Yue Yin, Yi Fan, Jun Zhao, Xiyu Mao, Jianbo Huang, Fan Yang, Ling Zhu

**Affiliations:** 1 College of Veterinary Medicine, Sichuan Agricultural University, Huimin Road 211, Weenjiang District, Chengdu, Sichuan China; 2 Key Laboratory of Animal Diseases and Human Health of Sichuan Province, Chengdu, Sichuan China

**Keywords:** *A. Pleuropneumoniae*, Dual RNA-seq, Immunological interactions, Immune response, Anaerobic metabolism, Immune evasion

## Abstract

**Background:**

The complexity of the pathogenic mechanism underlying the host immune response to *Actinobacillus pleuropneumonia* (*App*) makes the use of preventive measures difficult, and a more global view of the host-pathogen interactions and new insights into this process are urgently needed to reveal the pathogenic and immune mechanisms underlying *App* infection. Here, we infected specific pathogen-free *Mus musculus* with *App* serotype 7 by intranasal inoculation to construct an acute hemorrhagic pneumonia infection model and isolated the infected lungs for analysis of the interactions by dual RNA-seq.

**Results:**

Four cDNA libraries were constructed, and 2428 differentially expressed genes (DEGs) of the host and 333 DEGs of *App* were detected. The host DEGs were mainly enriched in inflammatory signaling pathways, such as the TLR, NLR, RLR, BCR and TCR signaling pathways, resulting in large-scale cytokine up-regulation and thereby yielding a cytokine cascade for anti-infection and lung damage. The majority of the up-regulated cytokines are involved in the IL-23/IL-17 cytokine-regulated network, which is crucial for host defense against bacterial infection. The DEGs of *App* were mainly related to the transport and metabolism of energy and materials. Most of these genes are metabolic genes involved in anaerobic metabolism and important for challenging the host and adapting to the anaerobic stress conditions observed in acute hemorrhagic pneumonia. Some of these genes, such as *adhE*, *dmsA*, and *aspA*, might be potential virulence genes. In addition, the up-regulation of genes associated with peptidoglycan and urease synthesis and the restriction of major virulence genes might be immune evasion strategies of *App*. The regulation of metabolic genes and major virulence genes indicate that the dominant antigens might differ during the infection process and that vaccines based on these antigens might allow establishment of a precise and targeted immune response during the early phase of infection.

**Conclusion:**

Through an analysis of transcriptional data by dual RNA-seq, our study presents a novel global view of the interactions of *App* with its host and provides a basis for further study.

**Electronic supplementary material:**

The online version of this article (10.1186/s12866-017-1105-4) contains supplementary material, which is available to authorized users.

## Background

*Actinobacillus pleuropneumonia* (*App*) can cause serious porcine respiratory diseases with high incidence and mortality rates. Fifteen serotypes of *App* have been detected, and these can cause acute hemorrhagic pneumonia and chronic interstitial pneumonia and have markedly affected the porcine industry [1]. Even with the optimization of intensive and large-scale breeding patterns and the development of vaccines for the disease, many districts have observed high morbidity rates due to *App* in recent years [2–4]. This disease is airborne, and the wild strains can easily break through the defense of a sound vaccine prevention and control system, resulting in rapid proliferation. Moreover, the infection and pathogenesis of *App* have become increasingly complex, particularly in cases of mixed infection [5]. An extensive body of clinical cases have reported that many wild strains show drug resistance to some routinely used antibiotics, such as tetracyclines [6], penicillins [7], erythromycin, streptomycin and tiamulin [8]. The evolution of drug resistance has resulted in the failure of drug treatment. Therefore, the development of efficient vaccines is an important undertaking, and to detect potential vaccine candidates, many virulence genes have been identified. Researchers have then analyzed the function of these genes, such as *HgbA* [9], *ureC* [10] and *apxIA* [11], through gene knockout to construct gene deletion mutants, providing more choices for subunit vaccine production.

The infection process is complex, and the interactions between a pathogen and its host are essential components of the infection. The pathogenesis and anti-infection mechanisms determine the development of the disease course. In fact, these mechanisms influence and restrict each other and ultimately yield differences in gene expression. Because the internal environment of the host shows marked differences from the in vitro environment, the expression of virulence factors by *App* is also notably different [12]; for example, *apxIVA* is differentially expressed in the two environments [13]. With the aim of determining the key point of the infection process and the differences between the environments, many experiments based on high-throughput sequencing have been conducted to identify differences in *App* gene expression between in vivo and in vitro conditions. Deslandes et al. [14] analyzed the differentially expressed genes (DEGs) of *App* during the acute phase of infection by microarray hybridization and identified three outer membrane proteins or lipoproteins, namely *fhaB* (*APL_0959*), *irp* (*APL_0919*) and *APL_0920*, as potential candidates for a protective cross-serotype vaccine. Klitgaard et al. [15] used DNA microarrays to analyze the potential strategies used by *App* for survival and persistence in the host. In addition, Zuo et al. [16] analyzed transcription in swine lung tissue by microarray hybridization to assess the regulation of the host defense response. However, the interactions between *App* and the host have not yet been elucidated. Brogaard et al. [17] selected 17 bacterial and 31 host genes from infected lungs and conducted the first high-throughput RT-qPCR analysis of the interactions between *App* and the host. Nevertheless, these techniques can detect only known genes and do not identify differentially expressed genes for which annotation information is lacking.

Based on these questions, we aimed to investigate the interaction between *App* and the host through RNA-seq. Different from microarray hybridization and high-throughput qPCR, RNA-seq can collect almost all transcription information from a specific sample(s) rather than monitor some specific genes. As a result, this technique can provide a global view of the interactions between the pathogen and the host [18, 19]. Moreover, we assessed transcription information for *App* and the host separately by dual RNA-seq and screened for potential causal relationships and interactions. In addition, a mouse model was selected for the study of *App* infection. Because specific pathogen-free (SPF) pigs are rare and costly and pigs in the field are easy affected by the pathogens found in the environment and vaccines, we had to select an animal model that is more easily available for this study. Compared with SD rat, guinea pig and rabbit, the SPF mouse is more susceptible [20], and the pathological changes caused by *App* are similar to those observed in typical cases of pigs naturally infected with *App* [21]; thus, a mouse model have been widely used for testing medicines and vaccines against *App*. Therefore, the present study also lays a foundation for the study of *App* infection in a mouse model and provides supporting data.

## Results

### Observations of symptoms and histopathology

In the current study, *Mus musculus* (*Mmu*) animals infected with *App* deteriorated over time and showed serious hemorrhagic pulmonary inflammation. Four hours after infection, the infected animals behaved differently from the control group, and these behavioral changes included anepithymia and depression. Six hours after infection, all of the infected animals exhibited depression, polypnea and disordered fur. In addition, 8 and 12 h after infection, two of the infected animals died, and another animal died 10 h post infection. In contrast, all of the animals in the control group were healthy and energetic.

**Fig. 1:**
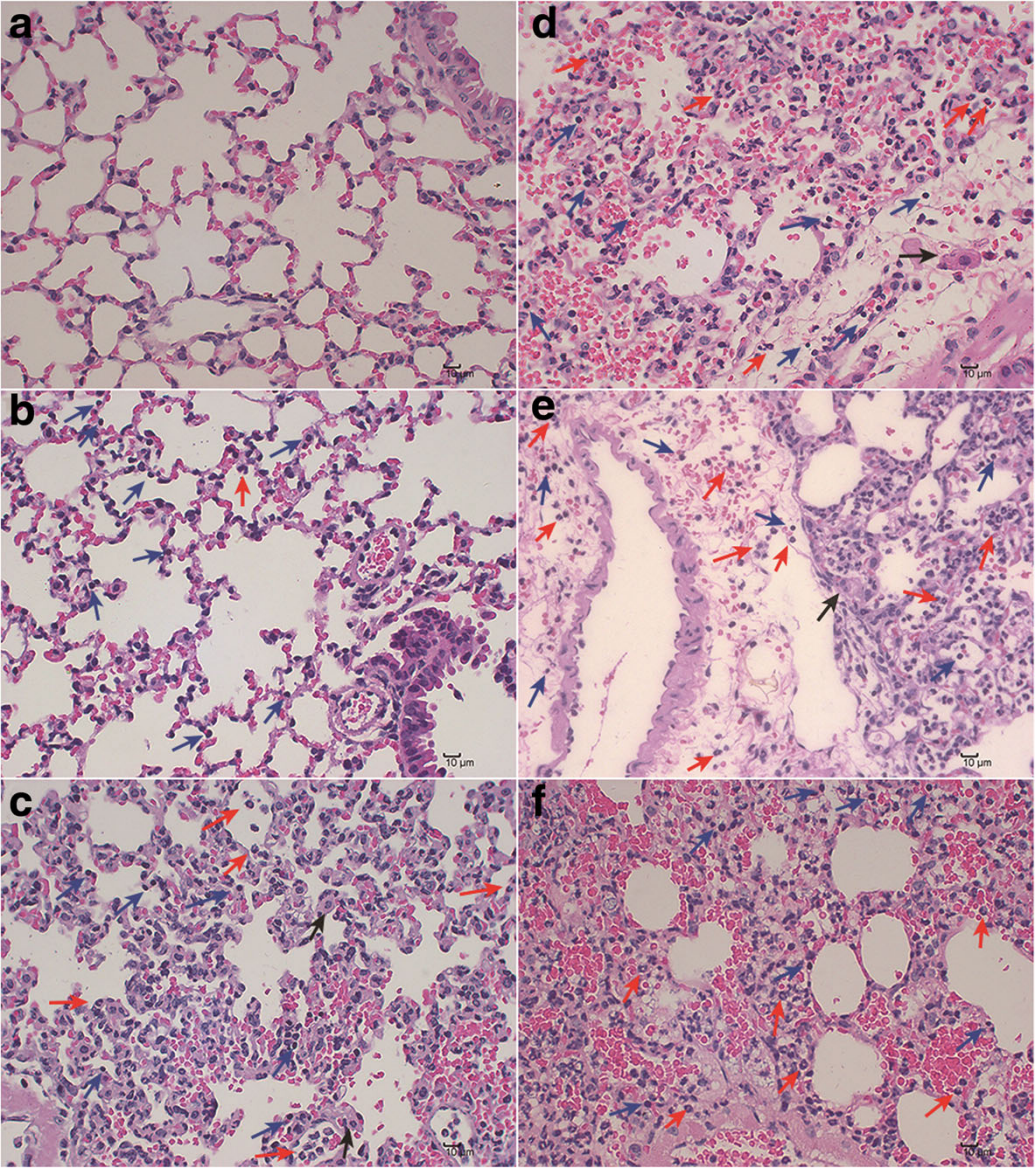
HE staining of lung tissue. Arrows: blue - leukomonocytes, red - neutrophils, black - macrophages. **a** Lung tissues from the control group. These tissues show a few RBCs from the trachea and bronchus in the interalveolar septa. Scale bar, 10 μm. **b**-**f** Lung tissues from the treatment groups. Scale bar, 10 μm. **b** Four hours after infection, interalveolar septa shows infiltration of inflammatory cells. **c** Six hours after infection, the interalveolar septa shows an increase of inflammatory cells, bleeding, and thickening. **d** Eight hours after infection, the interalveolar septa is filled with fibrin and cells, including leukomonocytes, neutrophils, macrophages and RBCs. **e** Ten hours after infection, a vein and interalveolar septa exhibit numerous neutrophils. **f** Twelve hours after infection, serious thickening of the interalveolar septa, with bleeding and infiltration of neutrophils and leukomonocytes, is observed

A histopathological analysis of the lungs by HE staining showed that all of the infected lungs were acutely inflamed and showed hyperemia, bleeding and infiltration of fibrin and inflammatory cells, including lymphocytes, macrophages, neutrophils and some eosinophilic granulocytes (Figs 1b-f). In addition, the infection led to degeneration of the alveolar septal cells and thickening of the interalveolar septa (Fig. 1b-f). These pathological changes were similar to those detected in a pig model by Brogaard et al. [17]. In summary, a hemorrhagic pulmonary inflammation model of *App* infection through intranasal inoculation was successfully established and used to obtain samples for dual RNA-seq.

### Transcriptomes of *Actinobacillus pleuropneumoniae* and *Mus musculus*

A comparison of the *App* content in all infected lungs detected by qRT-PCR was performed, and the mean Ct values of the five groups were 27.2, 27.27, 24.05, 28.88 and 29.42, respectively. Accordingly, five RNA samples obtained 8 h after infection showed a high content of *App*, with Ct values of 20.86, 22.09, 23.2, 24.56 and 25.61, and this time point was thus selected for sequencing. In addition, the RNA samples of bacterial cells and five healthy lungs were chosen as control groups for DEG identification.

**Fig. 2:**
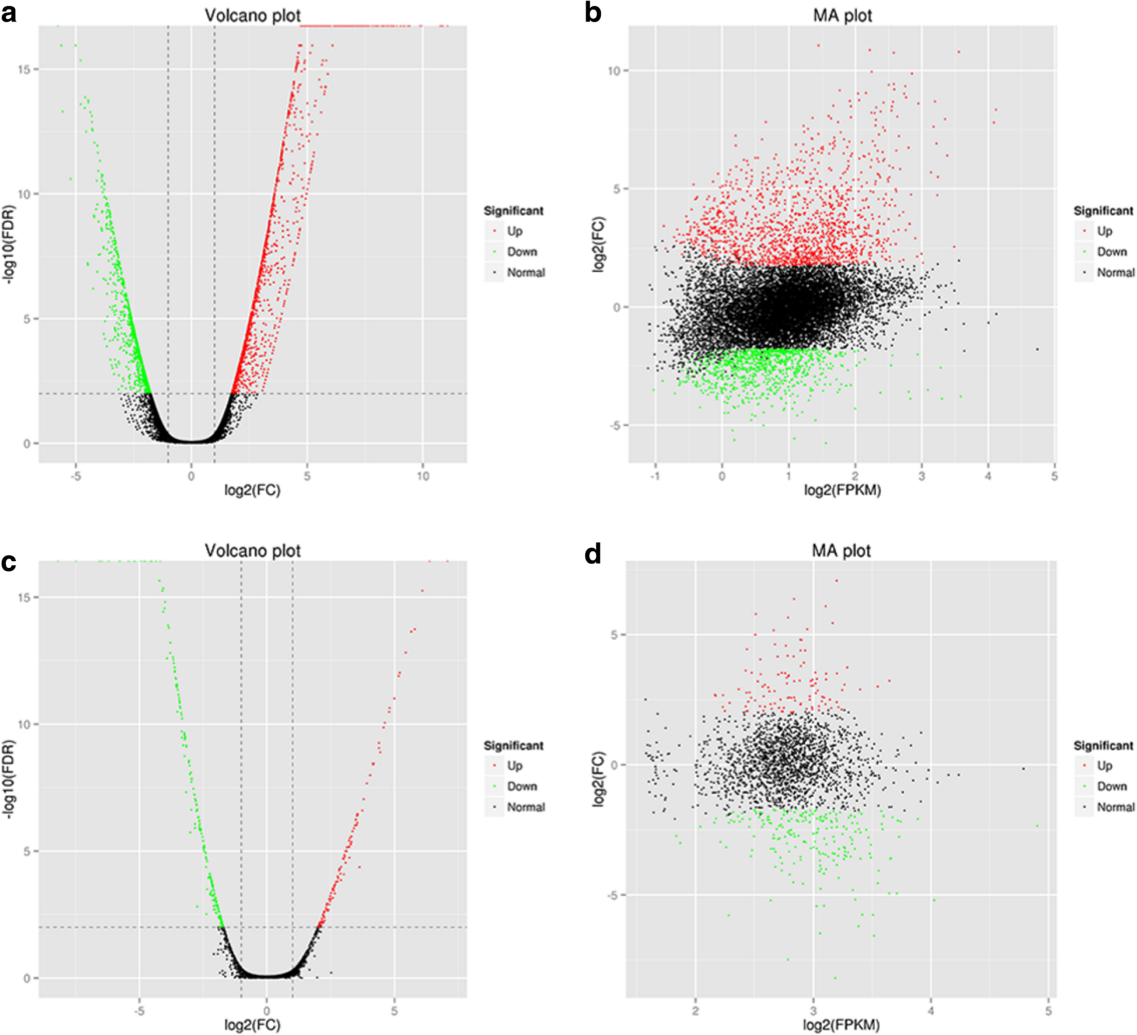
Overview on differences in gene expression. **a**, **b** DEGs of *Mmu*: the log_2_ (fold change) values of the up-regulated and down-regulated genes were in the intervals of [1.47, 11.04] and [−5.76, −1.75], respectively, and the FPKM, which is used to measure the level of transcription or gene expression, was in the interval of (−4, 32). **c**, **d** DEGs of *App*: the log_2_ (fold change) values of the up-regulated and down-regulated genes were in the intervals of [2.00, 7.07] and [−8.18, −1.70], respectively, and the FPKM was in the interval of (2, 32)

To construct libraries of the infected lungs, 67.68% of reads were mapped to the reference genome of *Mmu*, and 15.68% were mapped to *App*, which provided sufficient data for further analysis. For the healthy lungs and *App* libraries, the mapped rates were 85.01% and 97.99%, respectively (Table 1). DEGs (fold change ≥ 2 and FDR < 0.01) were defined using EB-Seq software (Fig. 2) and were annotated with the COG, GO, KEGG, Swiss-Prot and nr databases (Table 2). A total of 2428 DEGs were found in the infected lung tissue compared with healthy tissue, and of these, 1484 and 944 genes were up-regulated and down-regulated, respectively. A total of 333 DEGs of *App* were detected in the infected lungs compared with the in vitro conditions, and of these DEGs, 113 and 220 were up-regulated and down-regulated, respectively.

**Table 1:** RNA sequencing reads of four cDNA libraries

Sources^a^	Total Reads^b^	Libraries^c^	Mapping to *App* ^d^	Mapping to *Mmu* ^e^
Infected lungs	101,449,062(100.00%)	T01	15,911,177(15.68%)	
		T03		68,663,015(67.68%)
*App*	13,934,718(100.00%)	T02	13,654,587(97.99%)	
Healthy lungs	74,591,572(100%)	T04		63,411,879(85.01%)

^a^Sources of the genes

^b^Quantity of the reads (normalized to 100%)

^c^Names of libraries: “T01”, genes isolated from infected lungs and mapped to *App*; “T02”, genes isolated from *App* growing in culture media and mapped to *App*; “T03”, genes isolated from infected lungs and mapped to *Mmu*; “T04”, genes isolated from healthy lungs and mapped to *Mmu*

^d^Quantity of genes mapped to *App* and percentage of total reads

^e^Quantity of genes mapped to *Mmu* and percentage of total reads

**Table 2:** Statistics of annotated DEGs

DEGs^a^	Annotated databases^b^					
	COG	GO	KEGG	Swiss-Prot	nr	All
*Mmu*	650	2132	1407	2224	2354	2354
*App*	267	275	237	293	331	331

^a^DEGs of *Mmu* and *App*

^b^Quantity of DEGs annotated in the Clusters of Orthologous Groups (*COG*), Gene Ontology (*GO*), Kyoto Encyclopedia of Genes and Genomes (*KEGG*), Swiss-Prot and nr databases

**Fig. 3:**
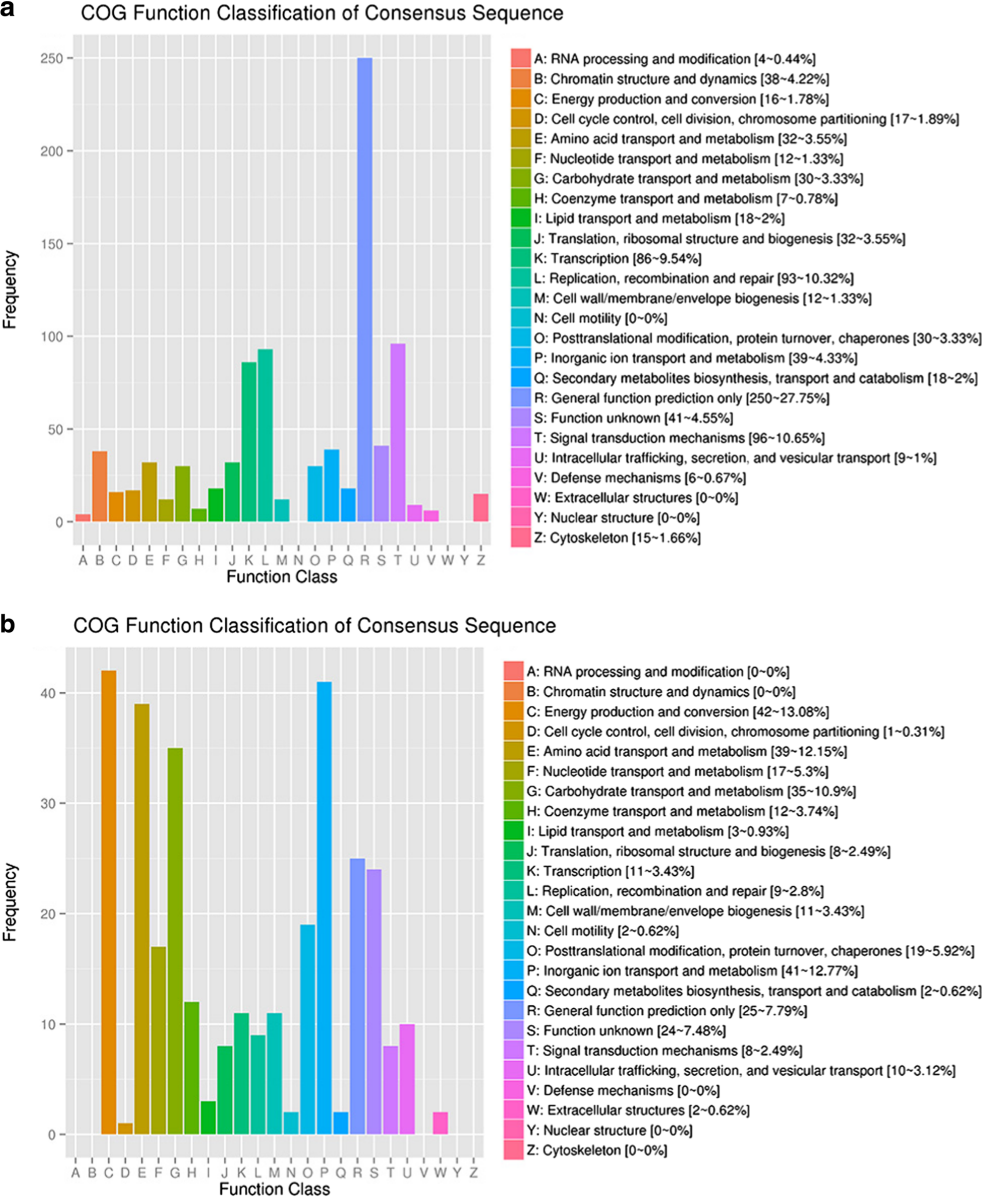
Function classification of the enriched DEGs in the COG database. **a** DEGs of *Mmu*. **b** DEGs of *App*

**Fig. 4:**
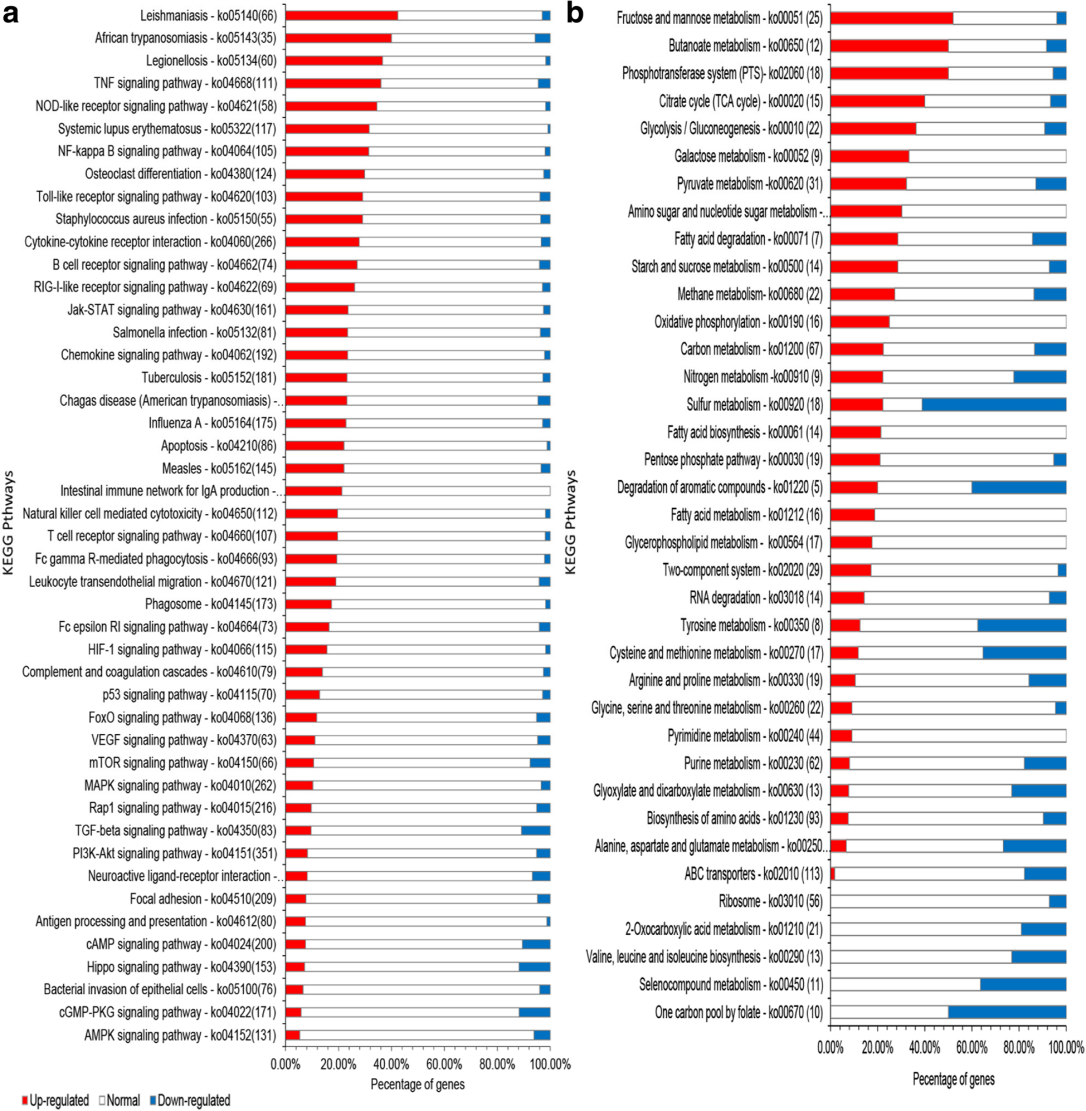
Overview of the transcriptional changes in KEGG pathways. **a** DEGs of *Mmu*. **b** DEGs of *App*

**Fig. 5:**
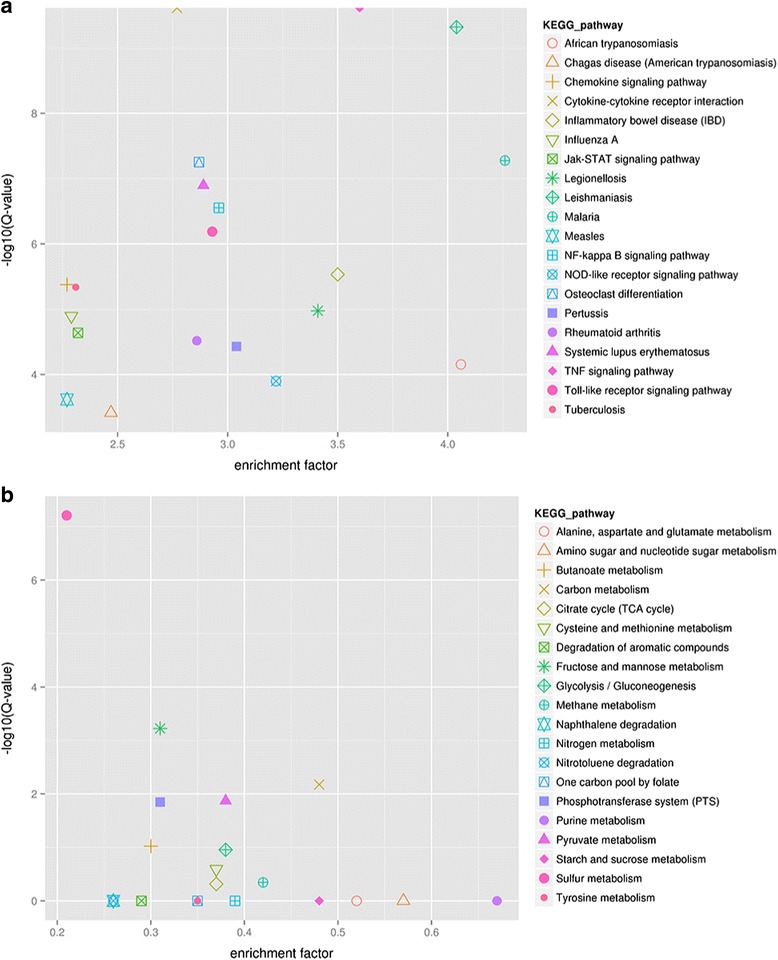
Twenty KEGG pathways with a lower enrichment factor and a higher Q value. **a** KEGG pathways of *Mmu*. **b** KEGG pathways of *App*

Enrichment analysis of the DEGs found in lung tissue showed that the genes were mainly associated with the “signal transduction mechanisms”, “replication, recombination and repair”, “transcription” and “general function prediction only” terms of the COG database (Fig. 3). To analyze the relationship between these DEGs and the immune system of the host, we annotated these genes according to KEGG pathways for statistics and analysis (Fig. 4). We identified 20 pathways that were enriched and shows statistically significant differences (Fig. 5). Notably, these pathways were mainly related to signal transduction mechanisms.

The DEGs of *App* were enriched in “energy production and conversion”, “inorganic ion transport and metabolism”, “amino acid transport and metabolism”, “carbohydrate transport and metabolism”, and other COG terms (Figs. 3 and 4) and in “carbon metabolism”, “fructose and mannose metabolism”, “pyruvate metabolism”, “amino sugar and nucleotide sugar metabolism”, and other KEGG pathways (Fig. 5). These genes represented the in vivo adaptation of the bacterial metabolism to the stresses present in the internal environment. Some metabolic genes also play key roles in pathogenicity.

**Fig. 6:**
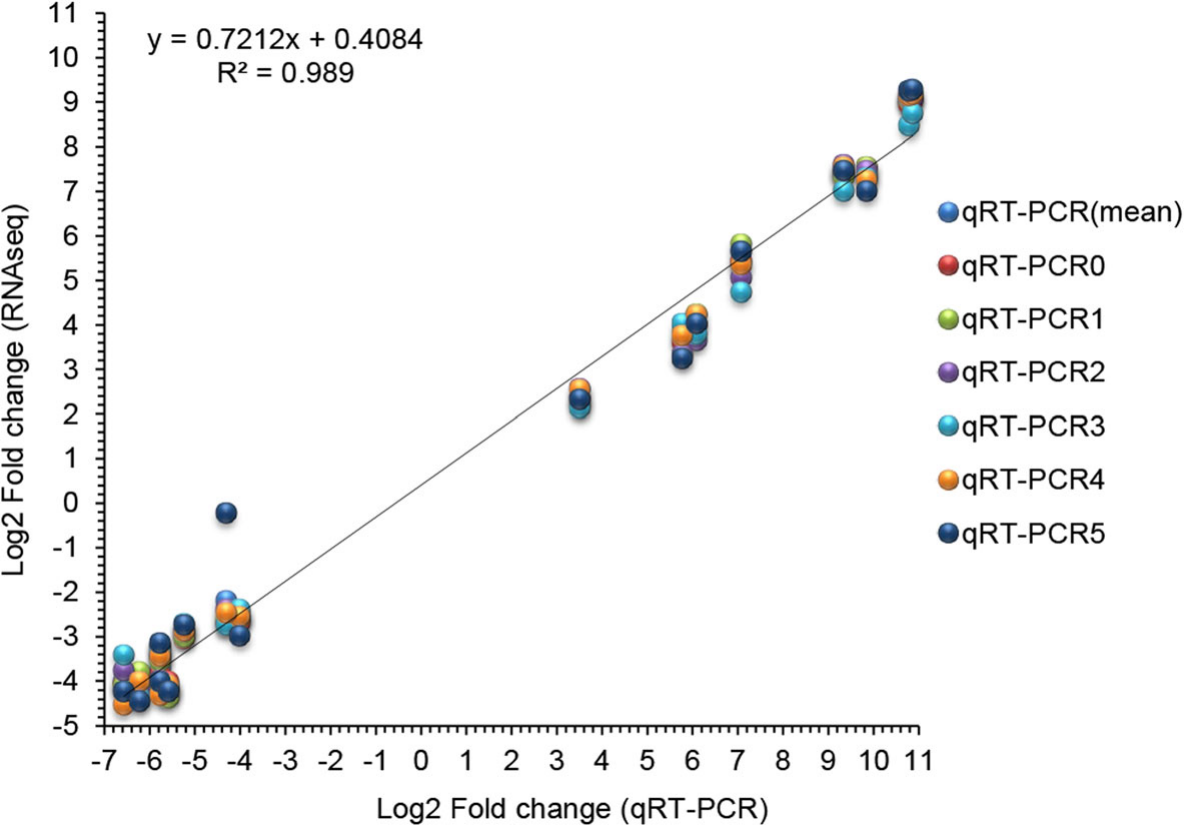
Comparison of relative expression levels measured by RNA-seq and qRT-PCR. qRT-PCR (mean): the mean of log_2_ (fold change) of six RNA samples. qRT-PCR0: the RNA sample used for sequencing. qRT-PCR 1–5: RNA samples mixed in equal parts for sequencing. Of the genes that were found to exhibit significant difference in expression by RNA-seq, the following were selected for detection by qRT-PCR: *adhE*, *dmsA*, *dmsB*, *fruk*, *malE*, *lam*b, *hiuH*, *metQ1* of *App*, and *cxcl2*, *csf3*, *ccl4*, *ccl3*, *Tpm3*, *60SrDNA*, *Csnk2a1* and *Hbα* of *Mmu*. A strong correlation (R^2^ = 0.989) in gene expression was found between the RNA-seq and qRT-PCR results

In addition, 18 genes showing high differences in expression, as identified by RNA sequencing, were selected to validate the gene expression findings obtained by qRT-PCR in order to confirm the reliability of the data. The five RNA samples from infected lungs and the sample used for sequencing were subjected to qRT-PCR assays, and their dispersion was analyzed. A strong correlation (R^2^ = 0.989) in gene expression was found between the RNA-seq and qRT-PCR results (Fig. 6).

### Anti-infection mechanism of the host associated with antigen recognition and immune response pathways

The analysis of the DEGs enriched in the host showed that the infection activated many antigen recognition and response pathways involved in the innate immune response, adaptive immune response and intercellular inflammatory signal transduction and directly showed the up-regulation of the genes of these pathways. In addition, many points of crosstalk were found among these signaling pathways (Additional file 1).

### Innate immune response: activation of pattern recognition receptors (PRRs)

Infection by *App* activated the PRR signaling pathways of the host, including the NOD-like receptor (NLR) signaling pathway (ko04621), the Toll-like receptor (TLR) signaling pathway (ko04620), the cytosolic DNA-sensing pathway (ko04623), Fc gamma R-mediated phagocytosis (ko04666), and caused up-regulation of *Tnf*, *Il1b*, *Il6*, *Il1*2, *MIP-1α*, *MIP-1β*, *IP-10*, *MIC*, *I-TAC*, *Ccl4*, *Ccl2* and *Cxcl10*. Interestingly, the RIG-I-like receptor (RLR) signaling pathway (ko04622), which is usually known for its antiviral activity, particularly in response to RNA viruses, was found to be activated in the present study, and caused up-regulation of *Tnf*, *Il12* and *IP10* for the adjustment of protein synthesis, growth arrest, dendritic cell activation, NK cell activation, CTL differentiation, antibody production and apoptosis.

### Innate immune response: activation of effector molecules and cells

The infection also activated many signaling pathways of effector molecules, including the TNF signaling pathway (ko04668), cytokine-cytokine receptor interaction (ko04060), the chemokine signaling pathway (ko04062) and the phagosome pathway (ko04145). Many cytokines can be regulated by the TNF signaling pathway, and the infection caused up-regulation of genes encoding leukocyte recruitment factors (*Ccl2*, *Ccl20*, *Cxcl1*, *Cxcl2*, *Cxcl3* and *Cxcl10*), leukocyte activation factors (*Csf1* and *Csf2*), surface receptors (*Fas*), inflammatory cytokines *(Il1b*, *Il6*, *Il15*, *Lif* and *Tnf*), intracellular signaling molecules (*Bcl3*, *Nfkbia*, *Socs3*, *Tnfaip3* and *Traf1*), transcription factors (*Fos* and *JunB*), an extracellular matrix remodeling factor (*Mmp9*), PRRS (*Nod2*), and cell adhesion factors (*Icam1*, *Sele* and *Vcam1*) and synthesis of inflammatory mediators (*Ptgs2*). The up-regulation of these proteins resulted in cytokine-cytokine receptor interaction. We determined that the up-regulated genes encoding chemokines included the CXC subfamily (*Cxcl9*, *Cxcl10*, *Cxcl11* and *Cxcl13*) and the CC subfamily (*Ccl20*, *Ccl19*, *Ccl2*, *Ccl4*, *Ccl3*, *Ccl7*, *Ccl8* and *Ccl11*), which further activated the chemokine signaling pathway and mediated a wider range of cytokine regulation.

In addition, both FcγR- and ER-mediated phagocytosis in the phagosome pathway (ko04145) were activated by the binding of the pathogen with phagocytosis-promoting receptors. *F-actin* on the membrane was up-regulated and promoted the internalization and formation of phagosomes with NADPH oxidase. In addition, under the action of the endoplasmic reticulum, the pathogen was degraded by H_2_O_2_ and bound to MHCI and MHCII for processing and presentation on the membrane. Furthermore, the MHCI and MHCII pathways in antigen processing and presentation (ko04612) were activated and promoted the recognition of the pathogen by the TCR of CD8 T cells, KIP of NK cells and TCR of CD4 T cells.

### Adaptive immune response: activation of humoral and cellular immunity

This study found that 27.03% of genes in the B cell receptor (BCR) signaling pathway (ko04662) were activated, promoting B cell ontogeny, immune response and immunoglobulin production. In addition, in the complement and coagulation cascade pathway (ko04610), the antibody-antigen complex activated genes encoding C2 and C5R1 by cascading effects. Finally, phagocyte recruitment and inflammation were stimulated, and the BCR signaling pathway was further activated. In addition, 19.63% of the genes in the TCR signaling pathway (ko04660) were up-regulated, and genes encoding membrane proteins, including *PD-1*, *CD45* and *CD36*, were activated, causing the overexpression of *Il10*, *Ifng*, *Csf2* and *Tnf* and promoting the proliferation, differentiation and immune response of T cells.

### Intercellular inflammatory signal transduction

The NF-κB signaling pathway (ko04064) is a key step of these signaling pathways, including the BCR, TCR, IL-1R, TNF-R1, RLR, TLR, CD40, RANK and LT-βR pathways, and regulated the transcription of multiple cytokines. We determined that 31.43% the genes involved in this pathway were up-regulated and caused up-regulation of *PIP1*, *c-IAP1/2*, *c-FLIP*, *TRAF1/2* and *A1/Bfl-1* for survival and of *COX2*, *MIP-1β* and *VCAM-1* for inflammation. In addition, the up-regulated expression of *Ifng*, *Il10*, *Il2/3* and *Il6*, which form part of the JAK-STAT signaling pathway (ko04630), activated the expression of *Jak* and *Shp1* by binding to receptors. With respect to the MAPK signaling pathway (ko04010), an important intercellular signal transduction pathway, 10.31% and 3.44% of its genes were found to be up-regulated and down-regulated, respectively. The JNK and p38 MAP kinase pathway was activated by the bacterial LPS, TNF, IL-1 and FASL TGFB. Some factors in the ERK5 pathway, such as serum, EGF and oxygen species, stimulated host cells and up-regulated *Nur77* for proliferation and differentiation.

### Pathogenic mechanism of *App* from bacterial metabolism in vivo

The annotation of the DEGs in the nr database helped show that most of them were metabolic genes, but their function can only be inferred by referring to other bacteria showing protein homology. In addition, we classified these in the COG database (Additional file 2).

### Energy production and conversion mechanisms

These significantly up-regulated genes were mainly enriched in “energy production and conversion”, a term in the COG classification, and were involved in anaerobic respiration, which is similar to the results of some previous studies [15, 22]. In fact, 67.50% of the genes associated with this term were up-regulated, many are involved in electron transport in respiration (GO:0009055). In addition, the majority of these genes encode hydrogenases and oxidoreductases, such as nitrite reductase (*nrfABCD*), periplasmic nitrate reductase (*napA*), trimethylamine-N-oxide reductase (*torYZ*), dimethylsulfoxide reductase (*dmsABCD*), fumarate reductase (*frdABCD*), and nitrate reductase A (*narP*), revealing the respiratory strategies of *App*, which involve the selection of nitrate, fumarate, dimethylsulfoxide (DMSO) and trimethylamine N-oxide (TMAO) as electron acceptors instead of oxygen. This finding indicates that the bacteria encountered an anaerobic environment during the invasion process. *App* cultured in isolated necrotic lung tissue or oxygen-deficient culture medium would regulate the expression of enzymes involved in respiratory mechanisms, which play an essential role in metabolic adaptations and virulence [23].

Genes such as *adhE*, *glpAC*, *napFGH*, mah and *hybABCDO* were highly up-regulated. The up-regulation was particularly obvious for *adhE*, which was annotated as a bifunctional acetaldehyde-CoA/alcohol dehydrogenase and participates in the biological processes of alcohol metabolism (GO:0006066), carbon utilization (GO:0015976), metal ion binding (GO:0046872) and oxidation-reduction processes (GO:0055114). In addition, proteins encoded by *NapF*, *hybA*, *glpC*, *napG* and *napH* have [4Fe-4S] dicluster domains and [4Fe-4S]-binding domains, and *glpA* also encodes a protein with a BFD-like [2Fe-2S]-binding domain. These proteins containing [Fe-S] diclusters are metalloenzymes with abundant biological functions, such as electron transfer and electron delocalization in respiration spin states and magnetism, catalysis in central metabolic pathways, gene regulation in response to the environment and protein structure stabilization [24, 25].

### Carbohydrate transport and metabolism

Carbohydrate metabolism is the major and direct source of energy for organisms. An analysis of carbohydrate transport and metabolism revealed that the proportion of up-regulated genes enriched in this term was 75.00%, and these genes were mainly involved in the metabolism of fructose, mannose, phosphoglycerate, triosephosphate, sucrose, glucose and galactose. Many of these activated enzymes belong to the phosphoenolpyruvate-dependent sugar phosphotransferase system (GO:0009401) and are involved in the biological processes of protein-N(PI)-phosphohistidine-sugar phosphotransferase activity (GO:0008982), integral membrane component (GO:0016021), and ATP binding (GO:0005524), among others.

The most significantly up-regulated genes, namely *pfkA* and *man XYZ*, were related to the metabolism of fructose and mannose. The *pfkA* gene, which in *Escherichia coli* encodes 6-phosphofructokinase, a key enzyme of glucose metabolism involved in growth under anaerobic conditions, is required in the Embden-Meyerhof pathway [26, 27]. In addition, *Escherichia coli* overexpressing the *manX*, *manY* and *manZ* genes show higher affinity to various organic solvents, but each these genes does not exert a significant individual effect [28]. These genes also help with glucose utilization, and the *manXYZ* mutation (***Δ*** *manXYZ*) decreases the utilization of glucose but increases that of xylose [29].

### Mechanism of inorganic ion transport metabolism

Many DEGs in the category “inorganic ion transport and metabolism” had commonalities in that they were connected to the transport and metabolism of iron (29.73%) and sulfate (35.14%). Genes associated with this term were mainly down-regulated, with a proportion of 72.97%. These significantly down-regulated genes included TonB-dependent receptors, catalase, formate/nitrite transporter, sulfur transferase and NLPA lipoprotein. Many of these genes are related to the virulence of *App*, which indicates that these proteins and enzymes are suppressed in the conflict between offense and defense.

### Amino acid transport and metabolism

The DEGs associated with this term were mainly down-regulated; in fact, the down-regulation rate was 80.65%. In addition, only five genes, namely *sdaC*, *sdaA*, *dmsD*, *ureC* and *ureB*, were up-regulated. Both *sdaC* and *sdaA* are involved in serine metabolism, which had the most significant differences in amino acid metabolism. It has been reported that serine is preferentially used in amino acid metabolism during growth in complex or defined media by *Campylobacter jejuni* [30], and this preference is due to three genes (*serABC*) of the bacteria [31], which are similar in terms of sequence to *Escherichia coli sdaA*, encoding an L-serine dehydratase. *SdaA* of *App* also encodes an L-serine dehydratase, with has the biological activity of degrading L-serine into pyruvate and ammonia to provide forms of carbon and nitrogen for the central metabolism. It also has a [4Fe-4S] cluster similar in structure to that of *Campylobacter jejuni*. The Fe^2+^ components of these Fe-S centers are considered to have the ability to reactivate L-serine dehydratases of some anaerobic bacteria under anaerobic conditions when these are inactivated by exposure to air. In addition, the sdaC protein is a serine transporter and septum formation initiator and belongs to the tryptophan/tyrosine permease family. *SdaC* in *Campylobacter jejuni* encodes a low-affinity L-serine-specific transporter and shows a similar structure to those of the *Escherichia coli sdaC* protein and members of the tryptophan/tyrosine permease family. Based on these data, we conjectured that *App* could have a preference for serine compared with other amino acids in vivo and that these L-serine dehydratases and serine transporter play vital roles in serine metabolism under anaerobic conditions; however, these inferences require further analyses.

Moreover, this study revealed that *ureC* and *ureB* were significantly up-regulated. These genes encode urease, which is important for the internal survival functions of the bacteria. Nina Baltes et al. compared the effects of an *App* mutant (***Δ*** *ureC*) and its parent strain on the immune system and found that the mutant strain revealed a higher number of *App*-specific B cells in the BALF, which suggested that the activity of *App* urease might cause inhibition/damage to the immune system of the host to aid in the persistence of the bacteria.

### Cell wall/membrane/envelope biogenesis

Genes involved in bacterial cell wall/membrane/envelope biogenesis were detected, and 10 of these were significantly regulated. Among them, *lrgB*, *alr*, *glmS*, *murC* and *murG* were up-regulated. The up-regulated genes *alr*, *murC* and *murG*, which encode the components of amino acid ligases, racemases and GTases, respectively, are involved in the synthesis of the peptidoglycan precursor [32]. Similarly, *glmS* encodes glucosamine-fructose-6-phosphate aminotransferase, and one of its catalytic products, *N*-acetylglucosamine, is an essential constituent of the bacterial peptidoglycan layer [33]. *IrgB* encodes a murein hydrolase regulator, and the protein also could regulate the growth of the bacterial cell wall. These murein hydrolases play a major role in the course of infection by releasing highly bioactive cell wall subcomponent autolysins [34].

### Identification of hypothetical proteins

Many DEGs had no available biological information or annotation details. All of the identified DEGs encoding hypothetical proteins, with the exception of *APP7_RS05355*, were down-regulated. A novel gene (novel 4) could be annotated in nr as an unknown protein of *Mannheimia succiniciproducens*.

## Discussion

In this study, a mouse model was established for the study of *App* infection. In addition, the results showed many similarities between mouse and pig in terms of clinical symptom, pathological changes of the lungs and the immune response, such as the activation of signaling pathways and the regulation of some cytokines. These findings show the applicability of the mouse model of *App* infection and provide a basis for further analysis. Our study presents a global view of the transcriptional profiling of *App* in the mouse host-lung model and further reveals the interactions between the bacteria and the host based on a large dataset of transcriptional information obtained by RNA-seq.

In addition, to capture many more transcripts of *App* in vivo from the complex transcriptional information arising from host interference, we selected to enrich the bacteria in the lung tissue through intranasal inoculation. We selected the infected lungs with the highest content of *App*, as detected by RT-qRCR, for sequencing, and increased the sequencing volume to ensure the accuracy and integrity of the results. We ultimately gained 15 Gb of clean data. These clean data were compared with the reference genome of *App* serotype 7, showing a comparison efficiency up to 15.68%, which is conducive for the subsequent analysis. As a result, we identified many significant DEGs that had never been previously reported. This study provides a basis and reference for further analysis of the physiological activity of *App* in vivo, particularly for the foundation of pathogenesis and immune evasion in *App.* The study also analyzed the transcriptional information of host lung tissue to reveal the anti-infection mechanism of the host.

### Activation of an anti-infection cytokine cascade

In the current study, the bacterial pathogens activated PRR signaling pathways, such as the TLR, NLR, and RLR signaling pathways. These bacterial pathogens include bacterial peptidoglycan, bacterial RNA, pore-forming toxin, flagellin, lipoarabinomannan, lipoprotein, lipopolysaccharide, and imidazoquinoline. The activation of these signaling pathways promoted the up-regulation of genes encoding cytokines, including granulocyte colony-stimulating factor, C-X-C motif chemokine, interleukin, C-C motif chemokine, tumor necrosis factor (TNF) and interferon gamma (IFNγ), which are listed in Additional file 3, and the fold-change in expression ranged 5 to 1871. The up-regulation of these cytokines on such a large-scale demonstrates the cytokine cascade involved in the host immune system.

Some of these cytokines are pro-inflammatory factors, such as IL-6, IL-1 and TNF, and their up-regulated expression increases the inflammatory response. In particular, the up-regulated expression of IL-1β and TNF activates the NF-κB signaling pathway and provides positive feedback to this pathway. Similarly, TNF activates the TNF signaling pathway and releases many cytokines, including those associated with leukocyte recruitment and activation factors, as well as inflammatory cytokines and transcription factors. The up-regulation of TNF also provides positive feedback to the pathway. *Tnf* and *Il1b*, which were found to be up-regulated in this study, could promote the transcription and expression of *Ptx3*, a humoral pattern recognition receptor and nonredundant component of humoral innate immunity for anti-infection [35]. It has been reported that IL-10, which is known for its anti-inflammatory activity, could also stimulate the secretion of PTX3 protein, presenting pro-inflammatory activity, whereas IFN-γ could inhibit the production of PTX3 [36]. Similarly, the acknowledged pro-inflammatory cytokines IL-6 and TNFα have also been reported to have anti-inflammatory abilities in the defense against *Mycobacterium tuberculosis* infection [37, 38]. The interactions of these cytokines are complex, and the role of anti-infection could be different in other bacteria, but the positive feedback of the signaling pathways could promote the observed cytokine cascade.

In addition, the IL-23/IL-17 axis was found to play a significant role in the host defense against bacterial infection in the present study. Cytokines such as IL-23, IL-17A, IL-17F, IL-1, IL-6, Csf2 and CXC are crucially regulated points of the IL-23/IL-17 axis and the genes encoding them were found to be highly up-regulated. IL-23 could regulate innate and adaptive immune responses and is produced by dendritic cells and macrophages rapidly when these are stimulated by bacterial pathogens. IL-23 and IL-1β activate Th17/Th_IL-17_ and other IL-17-producing cells, such as natural killer T (NKT) cells, which could recognize microbial glycosylceramides, a component of the cell wall of Gram-negative bacteria, to release IL-17, which could induce the secretion of G-CSF [39, 40]. As a result, the IL-23/IL-17/G-CSF pathway is activated to augment neutrophils for bacterial removal [41]. IL-23 also promotes the expression of pro-inflammatory cytokines, such as IL-1, IL-6 and TNF [42], and some inflammatory factors, such as Csf2 (GM-CSF), Ccl20, Ccl22, IL-1 and IL-23. Th17 cells, a key player in autoimmune diseases and antibiosis, are differentiated from naïve T cells (CD4+) stimulated by IL-6, TGF-β, IL-1β, IL-21 and IL-23 [43–46] and release IL-17A, IL-17F, IL-21 and IL-22. Together with TGF-β, IL-6 can induce the generation and maturity of Th17 cells and can inhibit TGF-β-mediated regulatory T cell (Treg) differentiation to regulate the Th17/Treg balance [47]. IL-22 has been reported to have an essential ability in recruiting neutrophils and soluble antibacterial factors to aid the response against bacterial infection [48], such as infection by *N. gonorrhoeae* [49] and *Citrobacter rodentium* [50]*.* Both IL-22 and IL-17 (IL-17R signaling) have been demonstrated to play crucial roles in maintaining the local control of Gram-negative pulmonary pathogens and regulate the secretion of CXC chemokines [51, 52], which are involved in the recruitment of neutrophils against bacterial pneumonia. Previous studies have confirmed that the IL-23/IL-17 axis of the host exerts antibacterial effects, and the present study demonstrates its involvement in the response to infection by *App*.

### Cytokine cascade could cause lung damage during infection

The infection with *App* caused acute bacterial pneumonia with infiltration by immune cells, including macrophages, neutrophils, eosinophils and lymphocytes, and released a large amount of inflammatory mediators through a cytokine cascade to defend against the infection. However, it also caused damage to pulmonary cells and tissues directly or indirectly. For instance, TNFα, which has the function of mediating components of the acute-phase response and stimulating granulocyte metabolism, has been proven to cause acute pulmonary vascular endothelial injury [53]. IL-6 is usually highly expressed when cells are stimulated by bacterial LPS, viruses and cytokines such as IL-1, IFN and TNFα, and the concentration of IL-6 and TNFα can reflect the severity of pneumonia [54, 55]. In the present study, the histopathological symptoms and high expression of *Il6*, *Il1*, *Ifng* and *Tnf* demonstrated that the host was suffering from serious acute bacterial pneumonia, which could be caused by the infection and the detected cytokine cascade.

### Anaerobic metabolism of *App* was activated in serious acute bacterial pneumonia

Serious bacterial pneumonia could lead to the disturbance of microcirculation and tissue blood perfusion, resulting in the failure of microcirculation and the onset of histanoxia. Many of the DEGs identified in this study are involved in anaerobic metabolism, which indicates that *App* in the infected lung was under anaerobic stress. Previous studies have demonstrated that genes such as *dmsA*, *aspA* and *hlyX* are essential for the virulence, inner survival and persistent existence of *App* in vivo by encoding dimethyl sulfoxide (DMSO) reductase [56], the aspartate ammonia lyase [57] and the transcriptional regulator FNR [58, 59], respectively. Many genes or enzymes involved in oxidative metabolism under anaerobic conditions are virulence genes or help regulate the expression of virulence genes. This study detected some genes that were significantly up-regulated but lacked profiles. Their details were few in *App* but numerous in other bacteria. For instance, *adhE* of *Escherichia coli* is essential for ethanol fermentation [60] and affects the virulence of bacteria under anaerobic respiration. By constructing an *Escherichia coli* mutant (*ΔadhE*), researchers found that *adhE* affects the expression of virulence genes, reduces the ability to adhere to host cells and activates identification of TLR-5 [61, 62]. This gene is also expressed in an aerobic environment [63], but its role is undefined. Genes such as *glpA*, *NapF*, *hybA*, *glpC*, *napG*, *napH*, *pfkA*, *CitT*, and *sdaA*, have been reported as important proteins in anaerobic metabolism, but their connection to bacterial virulence needs further research.

### In vivo immune evasion strategies of *App*

The bacterial cell wall and envelope constitute the first line of defense against attacks by the host immune system. In addition, the up-regulation of *alr*, *murC*, *murG* and *glmS* identified in this study promotes the biogenesis of peptidoglycan and helps maintain the cell shape [64]. Boneca et al. demonstrated that peptidoglycan modifications, including amidation, acetylation and glycosylation [65], could help *Listeria* gain resistance in the host by immune evasion [66], but this requires further study in *App*. In addition, genes encoding urease, which helps hydrolyze urea to ammonia and water, were found to be up-regulated, and ammonia could enhance neutrophil-dependent cell damage and cause inactivation of C4 of the complement cascade for immune evasion [67, 68].

Notably, the major virulence genes of *App* identified in the present study were down-regulated or showed no significant difference in expression. These include genes that encode RTX toxins, capsule proteins, outer membrane proteins and transferrin-binding proteins. Most of these proteins are present on the membrane and could be recognized by the host and eliminated. This result is similar to those obtained by Deslandes et al., who found that important virulence genes, including *cpx ABCD*, *apxI BCD*, *apxII AB* and *Tbp AB*, showed no significant difference in expression. By constructing gene deletion strains, these researchers found that the absence of capsule genes contribute to the adhesion of *App* in host cells [69]. The restricted expression of these genes might be a strategy of *App* for in vivo immune evasion.

### Hypothesis of the dominant antigens expressed in different phases

Based on the differential expression of *App* genes identified in our and other studies, we inferred that different and specific dominant antigens are expressed at different phases of *App* infection, and these could thus be considered vaccine candidates instead of or in addition to major virulence genes. In the early phase of infection, bacteria are adapting to the host environment, and a precise and targeted immune response would be advantageous to the rapid removal of the pathogen. Therefore, more studies on the transcriptional profiling of different infection phases should be performed to detect vaccine candidates.

## Methods

### Animal ethics statement

All animal procedures used in the present study were conducted in accordance with good animal practices as defined by the laboratory animal use license (Certificate No. SYXK (CHUAN) 2014–187). All work with mice was approved and supervised by the Committee on the Care and Use of Laboratory Animals of Sichuan and was conducted in the Animal Biotechnology Center of Sichuan Province at Sichuan Agricultural University. The animal interventions were performed in strict accordance with animal ethical standards. In addition, to limit any suffering, all mice used for lung collection were euthanized by rapid decapitation at the endpoint, which could also reduce the hemocytes in the tissues used for histopathological analysis.

### Animals and bacterial strains

The SPF KM mice (*Mus musculus,* provided by Chengdu Dashuo Biological Technology Co. Ltd.) used in the study were similar in age and closely related. They weighed 25–28 g and were housed in appropriate containment facilities with feed and water provided ad libitum.

*App* serotype 7 (CVCC265, preserved in China Institute of Veterinary Drug Control) isolated from pigs with acute pleuropneumonia was used for the infection study. The strain was cultured at 37 °C on tryptone soy broth (TSB) with 15 μg/ml NAD and 10% calf serum. When the bacterial strain reached the exponential phase in TSB, it was harvested at 4 °C by low-speed centrifugation (4000 rpm × 15 min). A portion of the bacterial cells were collected and rapidly frozen in liquid nitrogen for RNA-seq, and the rest of the cells were suspended with PBS to a final concentration of 2.69 × 10^10^ cfu viable bacteria/ml and used for infection.

### Infection studies

In the study, 35 SPF *Mmu* were infected with *App* serotype 7 by intranasal inoculation at a total dose of 5.38 × 10^9^ cfu (0.2 ml, 10 × LD_50_), and these mice constituted the treatment group. Twenty-five SPF *Mmu* served as the control group and were treated with PBS in the same manner. After infection, the two groups were housed separately in appropriate containment facilities in the laboratory, with feed and water provided ad libitum. In addition, their intake of food and water and their mental status were recorded once per hour.

The infection made the animals weaker, and the dyspnea worsened over time. To ensure that the infection successfully caused acute hemorrhagic pneumonia, their lungs were collected for histopathological observation. In addition, to limit their suffering and observed the typical pathological symptoms, the infected animals were euthanized by rapid decapitation at different time points until 12 h after infection and before their natural death. Seven animals from the treatment group were sacrificed 4 h, 6 h, 8 h, 10 h and 12 h post-infection, respectively, and their whole lungs were isolated. In addition, five animals from the control group were sacrificed in the same manner at the same time points.

From each whole lung, a section was rapidly frozen in liquid nitrogen for RNA isolation to estimate the content of the bacterial transcriptome, and the rest was fixed in 4% paraformaldehyde fixative solution to prepare paraffin sections and stained with HE for histopathological analysis.

### RNA isolation and reverse transcription

The total RNA from the infected lungs was extracted using an RNAiso Plus chloroform-isopropanol-ethanol extraction protocol. The RNA purity, concentration and integrity were measured using a Nano Drop spectrophotometer (Thermo Scientific), a Qubit 2.0 instrument (Invitrogen) and an Agilent 2100 Bioanalyzer (Agilent Technologies), respectively. The quality requirement was 2.0 ≥ A260/A280 ≥ 1.8 and RIN (RNA integrity number) > 5. Otherwise, a new extraction was performed. The resulting high-quality RNA was used for reverse transcription. All of the RNA samples from the lungs were diluted to 100 ng/μl with ddH_2_O, and 10 μg of each sample was used for reverse transcription with an RNA Library Prep Kit for Illumina (NEBNext® Ultra™). A qRT-PCR method (y = −3.313X + 40.228) based on the *16SrDNA* of *App* was performed to calculate the content of *App* in the RNA samples, and the samples with the five highest contents were selected for further RNA sequencing.

### Library preparation and RNA sequencing

The high-quality RNA samples were reverse transcribed and used for library construction. The prokaryotic rRNA was removed by probes showing complementarity to rRNA, and the rRNA-depleted RNA could then be enriched. The eukaryotic mRNA was directly enriched by magnetic beads with Oligo(dT) from the total RNA. The details of the library preparation are described in the instructions provided by the RNA Library Prep Kit for Illumina (NEBNext® Ultra™). For RNA sequencing, we used Illumina HiSeq2500, a high-throughput sequencing platform that can provide high-quality raw data (reads) with a base calling accuracy greater than 99.9%. Removal of the sequencing joints and primer sequences and filtering out low-quality data helped us achieve high-quality clean reads.

### Analysis of read mapping and gene expression

The clean reads were aligned against their reference genome by Bowtie and TopHat2 [70, 71]. The reference genomes of *App* and *Mmu* could be downloaded from the NCBI database with the ID numbers NC_010939.1 and GCA_000001635.7, respectively. The successfully aligned reads were considered mapped reads, and the rate of mapping (mapped reads divided by total reads) was used to evaluate the data reliability of the selected reference genome assembly. RNA sequencing showed high sensitivity in the detection of gene expression, and the level of transcription or gene expression was measured by RPKM, the coverage of which ranged from 10^−2^ to 10^4^ [72, 73].

### Screening and functional analysis of DEGs

DEGs obtained from the analysis of host genes in infected and healthy lungs (control) and *App* genes in in vivo and in vitro cultures (control) were defined using EBSeq software [74]. The Benjamini-Hochberg method was used for correcting multiple comparisons, and the DEGs were defined based on fold change ≥ 2 and FDR < 0.01. The functional annotation of DEGs was performed based on various databases, including the GO, COG, KEGG, Swiss-Prot, nr and Pfam databases.

### Comparison of RNA-seq and qRT-PCR results

The RNA sequencing results for transcript profiling were verified by qRT-PCR using SYBR® Premix Ex Taq™ II (2×) (TaKaRa) and the same RNA samples that were used for RNA sequencing. The qRT-PCRs were conducted on a BIO-RAD CFX, and each reaction was performed in a total volume of 25 μl containing 12.5 μl of SYBR® Premix Ex Taq™ II (2×), 2 μl of the cDNA template, 1 μl of each primer (0.5–2.5 μl), and 8.5 μl of ddH_2_O. All of the reactions were performed in triplicate, and the reaction system was optimized by adjusting the concentrations of the primers and template.

The primers for qRT-PCRs were designed by Primer Premier 5.0 and Primer-BLAST (Additional file 4). The thermal cycling conditions were as follows: 95 °C for 20 s, 40 cycles of 95 °C for 10 s, 55 °C for 30 s, and 72 °C for 30 s, with fluorescence detection at the end of each cycle, and a melting curve analysis (from 60 °C to 95 °C, increasing at a rate of 1 °C/3 s) to ensure that each reaction amplified a single and specific product. The qRT-PCRs showed high amplification efficiency (between 95% and 105%). Two reference genes, the bacterial 16S ribosomal and GADPH genes of *Mmu*, which showed only a slight variation in the RNA-seq analysis, were used for data normalization.

## Conclusions

To obtain insights into the interactions between *App* and its host and provide a profile of the pathogenesis and adaptive metabolism of *App* and the immune and anti-infection responses of the host, an infection model of acute bacterial pneumonia in SPF *Mmu* was established. The infected lungs were isolated for dual RNA-seq, and the transcriptional differences of *App* and *Mmu* between infection and non-infection conditions were revealed. In total, 2428 *Mmu* genes and 333 *App* genes were identified as DEGs. The identified host DEGs were mainly up-regulated and activated many immune signaling pathways, such as the TLR, NLR, RLR, RIG-I, BCR, and TCR signaling pathways, and caused up-regulation of *Csf2*, *Cxcl2*, *Il6*, *Ccl*4, *Il1b*, *Il23α*, *Il1α*, *Il10*, *Il17f*, *Il17a*, *Tnf*, and *Ifng*, among other cytokines, leading to an anti-infection cytokine cascade and causing lung damage. Most of these cytokines are involved in the IL-23/IL-17 cytokine interaction network, which might play a significant role for the host in *App* infection. The DEGs of *App* were mainly involved in anaerobic metabolism. These include *adhE*, a metabolic gene that has been proven to be virulence gene in *Escherichia coli*, *sdaC*, *dmsA*, *dmsB*, *torY*, *torZ*, and *glpA*. Furthermore, *App* was found to exhibit various strategies for immune evasion, such as enhancing the cell wall and damaging immune cells by synthesizing urease. The strategies regarding the restriction of major virulence genes provide novel vaccine candidates; specifically, different infection phases might be characterized by different dominant antigens, and these dominant antigens might aid the establishment of a precise and targeted immune response during the early phase of infection. These findings are meaningful for the study of *App* infection, but the differences between mice and pig exist indeed, so more verifications to present their roles in pigs need to be performed in the further study.

## Additional files

Additional file 1: Activated signaling pathways involved in the inflammatory response. These inflammatory signaling pathways were regulated after infection and directly present based on the regulation of their genes, including up-regulation and down-regulation. (PDF 168 kb)

Additional file 2: DEGs of *App* classified by COG terms and annotated in the nr database. These DEGs of *App* were mainly metabolic genes and annotated in the nr database. They were classified into COG terms to clearly analyze their functions. (PDF 200 kb)

Additional file 3: Differentially expressed cytokines of the host. These DEGs of the host could be annotated in the nr database as cytokines, and the log_2_ (fold change) values were used to present their expression level. (PDF 170 kb)

Additional file 4: Gene-specific primers for qRT-PCR. Significant DEGs of *App* and *Mmu* were selected to test the RNA-seq results. The DEGs of *App* included *adhE*, *dmsA*, *dmsB*, *fruK*, *malE*, *lamB*, *hiuH*, *MetQ1*, and the DEGs of *Mmu* included *Cxcl2*, *Csf3*, *Ccl4*, *Ccl3*, *Tpm3*, *60SrDNA*, *Csnk2a1*, *Hbα*. *16SrDNA* and *GAPDH* were used as the reference genes of *App* and *Mmu*, respectively. (PDF 182 kb)

## Abbreviations

*App*: *Actinobacillus pleuropneumonia*
BCR: B cell receptor
COG: Clusters of Orthologous Groups
CTL: Cytotoxic lymphocyte
DEGs: Differentially expressed genes
FC: Fold change
FDR: False discovery rate
FPKM: Fragments per kilobase of exon model per million mapped reads
GO: Gene Ontology
IL: Interleukin
KEGG: Kyoto Encyclopedia of Genes and Genomes
Mmu: Mus musculus
NK: Natural killer cell
NLR: NOD-like receptor
PAMPs: Pathogen-associated molecular patterns
PRRs: Pattern recognition receptors
RLR: RIG-I-like receptor
SPF: Specific pathogen-free
TCR: T cell receptor
TLR: Toll-like receptors
