# Supplementary material for: Transcript profiling of the immunological interactions between Actinobacillus pleuropneumoniae serotype 7 and the host by dual RNA-seq
Source: BMC Microbiol. 2017 Sep 12;17:193. doi: 10.1186/s12866-017-1105-4 (PMC5596872; doi:10.1186/s12866-017-1105-4)
Supplement: Supplementary file 1 — Activated signaling pathways involved in the inflammatory response. These inflammatory signaling pathways were regulated after infection and directly present based on the regulation of their genes, including up-regulation and down-regulation. (PDF 168 kb) [file 12866_2017_1105_MOESM1_ESM.pdf]

Additional file 1: Activated signaling pathways involved in inflammatory response.

KEGG pathways	Ko ID	Regulation of DEGs	
		**Up-regulated**	**Down-regulated**
NLR signaling pathway	ko04621	*Nod2, Rip2, Ci1/2, A20, IκB, NF-κB, Casp1, Nalp3, Pyrin, Pstpip1, Ipaf, Naip5, Il1b, Ccl2, Tnfα, Il6*	*Erk*
TLR signaling pathway	ko04620	*Tlr1, Tlr2, Tlr6, Tlr4, Tlr7/8, CD14, MyD88, P13K, Rip1, p105, Tp12, IκB, NF-κB, Irf5, Irf7, AP-1, TNF, Il1b, Il6, Il12, Mip-1α, Mip-1β, CD40, CD80, CD86, IP-10, Mig, I-Tac*	*Tlr5, Mkk3/6, Erk*
RLRsignaling pathway	ko04622	*RIG-I, Cyld, Rnf125, Isg15, Lgp2, Mda5, Mita, Tank, Nap1, Rip1, Ddx3x, Irf7, IκB, NF-κB, Tnfα, Il12, IP10*	*Ips1, Sntbad*
Cytosolic DNA-sensing pathway	ko04623	*RIG-I, cGas, Sting, Rip1, p202, Casp1, IκB, NF-κB, Irf7, Il1b, Il23, Il6, Ccl4, Cxcl10*	*Ips1*
FcγR-mediated phagocytosis	ko04666	*FcγRrIIb, CD45, FcγRi, Src, Syk, Pi3k, Vav, Dock180, Sphk, Rac, Wasp, Vasp, Marcks, cPkc, p47phox*	*Erk1/2, Pi3k*
Chemokine signaling pathway	ko04062	*Chemokine, Chemokine R, Grk, Jak2/3, Gai, Src, Pi3k, Stat, Dock2, Itk, Vav, p47phox, Wasp, IκB, NF-κB,*	*Erk1/2*
BCR signaling pathway	ko04662	*Shp1, Lyn, Vav, Syk, Bcap, Pi3k, Bam32, Rac, Bcl-10, Malt1, IκB, NF-κB, Ap1*	*Igα, Pi3k, Erk*
TCR signaling pathway	ko04660	*Pd1, CD45, CD38, Shp1, Slp-76, Vav, Itk, Pi3k, Bcl-10, Malt1, Cot, IκB, NF-κB, Ap1, Il10, Ifng, Csf2, Tnf*	*Erk, Pi3k*
Complement and coagulation cascades	ko04610	*F3, F10, F5, Plat, P13, Serpinei, Plaur, Bdkr, C2, C5r1*	*Kng, Plg*
Antigen processing and presentation	ko04612	*Ifng, Tnf, MhcI, MhcII, Tap1/2*	*Nfy*
NF-κB signaling pathway	ko04064	*Il1b, Tnf, CD14, TLR4, CD40, Rank, Light, Syk, Lyn, My88, Rip1, RIG-I, Ciap1/2, cIAP1/2, Malt1, IκB, p50, p100, RelB, p52, c-IAP1/2, c-Flip, Traf1/2, A1/Bf1, A20, IκBα, Cox2, Mip1β, Vcam1, Elc, Icam*	*Ck2, Pidd*
Jak-STAT signaling pathway	ko04630	*INF/Il10, Il2/3, Il6, Cytokine R, Jak, Shp1, Stat, Pi3k, Socs, Pim1, c-Myc, Spred, Sprouty*	*Cytokine R, Plas, Pi3k, Spred*
MAPK signaling pathway	ko04010	*Tnf, Il1, Il1R, Fas, CD14, Gadd45, Daxx, Cdc42/Rac, Tpl2/Cot, Ask2, Tao1/2, Mkp, Mafkafk, Creb, Nur77, Gaplm, Pkc, NF-κB, c-Myc, c-fos*	*Cacn, Fgf, Fgfr, Tgfb, Lzk, Mkk6, Ptp, Erk,*
Phagosome	ko04145	*F-action, coronin, Tua, Tubb, MhcI, MhcII, Tap, Fcyr, Cr3, aMβ2, Tsp, collectins, Tlr2, Tlr4, Tlr6, CD14, Mr, Sra1, Marco, Lox1, gp91, p47phox, p67phox, p40phox.*	*Tubb, vATPase, Mr*
TNF signaling pathway	ko04668	*cIAP1/2, Rip1, Tpl2, NF-κB, IκB, Mlkl, c-Flip, c/Ebpβ, Creb, Tnf/Lla, Tnfr2, Traf1, Pi3k, Rip, Ccl2, Ccl20, Cxcl1, Cxcl2, Cxcl3, Csf1, Csf2, Fas, Il1b, Il6, Il15, Lif, Tnf, Bcl3, Nfkbia, Socs3, Tnfaip3, Traf1, Fos, Junb, Mmp9, Nod2, Icam1, Sele, Vcam1, Ptgs2.*	*Pi3k, Mkk3/6, Erk1/2, Creb*
Cytokine-cytokine receptor interaction	ko04060	*Cxcl9, Cxcl10, Cxcl11, Cxcl13, Il8Rb, Il8Ra, CxcR7; Ccl20, Ccl19, Ccl2, Ccl4, Ccl3, Ccl7, Ccl8, Ccl11, Ccr9, Ccr7, Ccr1, Ccr3; Il6, Csm, Osmr, Lif, Csf3, Csf3r; Il4r, Il13ra1; Il12, Il12rb2, Il23a; Csf2, Csf2rb; Il7, Il7r, Il15, Il15ra, Tslp; Csf1; Ifng; Il10, Il10Ra, Il22; Tnfsf15, Sf11b, Sf11a, Sf12a, Tnf, Sf1b, Tnfsf14, Fas, CD40, Tnfsf8, Sf8, Tnfsf9, Sf9, SF19L; Il17a, Il17r; Il1a, Il1b, Il1rap, Il1r2, Il18rap*	*Cx3cr1; Epor; Kdr; Sf19; Tgfb3, Acvr2b, Amhr2, Bmpr1b, Bmp7*
