# Supplementary material for: Transcript profiling of the immunological interactions between Actinobacillus pleuropneumoniae serotype 7 and the host by dual RNA-seq
Source: BMC Microbiol. 2017 Sep 12;17:193. doi: 10.1186/s12866-017-1105-4 (PMC5596872; doi:10.1186/s12866-017-1105-4)
Supplement: Supplementary file 2 — DEGs of App classified by COG terms and annotated in the nr database. These DEGs of App were mainly metabolic genes and annotated in the nr database. They were classified into COG terms to clearly analyze their functions. (PDF 200 kb) [file 12866_2017_1105_MOESM2_ESM.pdf]

Additional file 2: DEGs of App classified in COG terms and annotated in nr database.

Gene name[a]	Locus tag[b]	log2FC[c]	Annotation[d]
**Energy production and conversion**			
adhE	APP7_RS05435	7.07794033315575	bifunctional acetaldehyde-CoA/alcohol dehydrogenase
dmsA	APP7_RS09005	6.09390636117657	dimethyl sulfoxide reductase subunit A
dmsB	APP7_RS09010	5.79570805569731	dimethyl sulfoxide reductase subunit B
torY	APP7_RS03715	5.65697034202674	cytochrome C nitrate reductase
torZ	APP7_RS03710	5.44331095039149	trimethylamine N-oxide reductase I catalytic subunit
glpA	APP7_RS02055	5.20690399367382	sn-glycerol-3-phosphate dehydrogenase subunit A
NapF	APP7_RS09025	4.43899324032373	hypothetical protein
mdh	APP7_RS06895	4.39114833022603	malate dehydrogenase
hybO	APP7_RS07075	4.183534118407	hydrogenase 2 small subunit
hybA	APP7_RS07080	3.5383005611253	putative hydrogenase 2 b cytochrome subunit
frdA	APP7_RS08205	3.52032862835459	fumarate reductase flavoprotein subunit
frdB	APP7_RS08200	3.44635298832695	fumarate reductase iron-sulfur subunit
pflB	APP7_RS05560	3.23296696714982	keto-acid formate acetyltransferase
glpC	APP7_RS02065	3.21837040926207	sn-glycerol-3-phosphate dehydrogenase subunit C
fumC	APP7_RS09620	3.08264586112048	fumarate hydratase
HI_1278	APP7_RS06385	-3.67804157468297	NAD(P)H nitroreductase
dld	APP7_RS03705	-4.34354548679215	D-lactate dehydrogenase
aldA	APP7_RS10955	-4.94045713112859	aldehyde dehydrogenase
lctP	APP7_RS02695	-5.76752838319845	lactate permease
**Carbohydrate transport and metabolism**			
pfkA	APP7_RS06015	4.79517143112524	6-phosphofructokinase
manY	APP7_RS08950	4.62315844579669	PTS mannose transporter subunit IIC
manX	APP7_RS08955	4.57099767835288	PTS mannose transporter subunit IIAB
manZ	APP7_RS08945	4.15152812199931	PTS mannose transporter subunit IID
pgk	APP7_RS06625	3.74223604876696	phosphoglycerate kinase
fruK	APP7_RS01790	3.52595205562162	1-phosphofructokinase
dhaL	APP7_RS00435	3.48723683388004	dihydroxyacetone kinase subunit DhaL
tpiA	APP7_RS10525	3.30897488354780	triosephosphate isomerase
fruA	APP7_RS01785	3.30682106451202	PTS fructose transporter subunit IIBC
manX	APP7_RS07450	3.28680122148373	PTS mannose transporter subunit IIAB
manY	APP7_RS07455	3.27521359282467	PTS mannose transporter subunit IIC
fruB	APP7_RS01795	3.04435865582476	PTS fructose transporter subunit IIA
eno	APP7_RS05960	3.01007982706774	enolase
fbp	APP7_RS07515	-3.35757922536416	fructose-1,6-bisphosphatase
malQ	APP7_RS06575	-3.39276862446776	4-alpha-glucanotransferase
malK	APP7_RS06555	-5.42092692104977	sugar ABC transporter ATP-binding protein
--	APP7_RS08850	-6.20711900255535	maltoporin
malE	APP7_RS06560	-6.57213364704286	sugar ABC transporter substrate-binding protein
**Inorganic ion transport and metabolism**			
citT	APP7_RS06200	4.80736354056664	Anion transporter
yvdB	APP7_RS06315	3.91920326120356	membrane protein

yfeX	APP7_RS06230	3.47491959669265	deferrochelatase/peroxidase YfeX
nrfD	APP7_RS00535	3.13218249654909	formate-dependent nitrite reductase subunit NrfD
nrfA	APP7_RS00520	3.12839499232311	cytochrome C nitrite reductase subunit c552
metI	APP7_RS04940	-3.15446496144941	methionine ABC transporter permease
cysN	APP7_RS10105	-3.16180054023841	sulfate adenylyltransferase
sbp	APP7_RS10125	-3.33021687088162	ABC transporter permease
metQ2	APP7_RS04935	-3.51608538993932	membrane protein
hugZ	APP7_RS05620	-3.56735499346002	hypothetical protein
HI_0608	APP7_RS06640	-3.60456021887598	transporter
cysJ	APP7_RS10100	-3.67004197624025	sulfite reductase
metQ1	APP7_RS04930	-3.99055963951199	membrane protein
pspE	APP7_RS06835	-4.5513733552095	sulfurtransferase
yrhG	APP7_RS10395	-4.57165118047258	Transporter
katA	APP7_RS05295	-4.81774189342019	catalase
hxuC	APP7_RS06910	-5.17722786959602	TonB-dependent receptor
**Amino acid transport and metabolism**			
sdaC	APP7_RS04660	6.37353039562096	septum formation initiator
sdaA	APP7_RS04665	4.39105425335854	serine dehydratase
glpB	APP7_RS02060	4.16076549395075	glycerol-3-phosphate dehydrogenase subunit B
ureC	APP7_RS08705	2.09672485363992	urease subunit alpha
ureB	APP7_RS08710	2.07303394051554	MULTISPECIES: urease subunit beta
yxjG	APP7_RS04645	-3.06848037026207	methionine synthase
occM	APP7_RS09100	-3.22430449338009	amino acid ABC transporter permease
ybgG	APP7_RS05785	-3.53480263881179	homocysteine S-methyltransferase
yxjG	APP7_RS01665	-3.61557038325985	5-methyltetrahydropteroyltriglutamate--homocysteine methyltransferase
nikA	APP7_RS00145	-3.61871851698151	ABC transporter substrate-binding protein
hbpA	APP7_RS04655	-3.84514389670273	peptide ABC transporter substrate-binding protein
yecS	APP7_RS09095	-4.05630379333911	amino acid ABC transporter permease
metX	APP7_RS02655	-4.76763404824823	homoserine O-acetyltransferase
metE	APP7_RS06195	-4.93936291238210	5-methyltetrahydropteroyltriglutamate--homocysteine methyltransferase
metF	APP7_RS04650	-6.48008196959430	5,10-methylenetetrahydrofolate reductase
**Cell wall/membrane/envelope biogenesis**			
HI_1298	APP7_RS04285	2.63513767482871	murein hydrolase regulator LrgB
alr	APP7_RS01295	2.57809337051588	alanine racemase
glmS	APP7_RS08780	2.21897351070532	glucosamine--fructose-6-phosphate aminotransferase
murC	APP7_RS00095	2.13597920345964	UDP-N-acetylmuramate--alanine ligase
murG	APP7_RS00090	2.03166459944423	UDP-diphospho-muramoylpentapeptide beta-N-acetylglucosaminyltransferase
apxID	APP7_RS07480	-2.02557125946843	hemolysin D
mscL	APP7_RS08585	-3.42916405861492	large conductance mechanosensitive channel protein MscL
PM0998	APP7_RS09915	-3.58404238960335	hypothetical protein
**Hypothetical protein encoded by DEGs**			
--[e]	APP7_RS05355	3.62649874814625	hypothetical protein

--	APP7_RS09290	-2.01314240456335	hypothetical protein
--	APP7_RS04300	-2.06210829306476	hypothetical protein
--	APP7_RS10040	-2.08212330431234	hypothetical protein
--	APP7_RS03645	-2.1154132837973	hypothetical protein
--	APP7_RS03245	-2.12157561322476	hypothetical protein
--	APP7_RS04120	-2.24669375422653	hypothetical protein
--	APP7_RS06950	-2.27135672845264	hypothetical protein
--	APP7_RS10265	-2.34171430641395	hypothetical protein
--	Novel_4	-2.3417852416682	Unknown[f]
--	APP7_RS01930	-2.39289578436922	hypothetical protein
--	APP7_RS05875	-2.76145956189759	hypothetical protein
--	APP7_RS05870	-2.93601525765115	hypothetical protein
--	APP7_RS07000	-2.96256984995383	hypothetical protein
--	APP7_RS04980	-3.34502806759688	hypothetical protein

[a] Gene name, annotated in Swissprot and nr, and nr was priority.

[b] Locus tag from Actinobacillus pleuropneumoniae serovar 7 str. AP76.

[C] Numbers with "-" were down-regulated and others were up-regulated.

[d] Annotations of Genes in nr.

[e] "--" in the table were genes without gene name annotated in Swissprot or nr.

[f] A novel gene similar to Mannheimia succiniciproducens MBEL55E.
