# Supplementary material for: Transcript profiling of the immunological interactions between Actinobacillus pleuropneumoniae serotype 7 and the host by dual RNA-seq
Source: BMC Microbiol. 2017 Sep 12;17:193. doi: 10.1186/s12866-017-1105-4 (PMC5596872; doi:10.1186/s12866-017-1105-4)
Supplement: Supplementary file 3 — Differentially expressed cytokines of the host. These DEGs of the host could be annotated in the nr database as cytokines, and the log2 (fold change) values were used to present their expression level. (PDF 170 kb) [file 12866_2017_1105_MOESM3_ESM.pdf]

Additional file 3: Differentially expressed cytokines of host.

Gene ID	Gene Name	Log2FC	Nr_annotation
ENSMUSG00000038067	*Csf3*	10.87	Granulocyte colony-stimulating factor precursor
ENSMUSG00000058427	*Cxcl2*	10.79	C-X-C motif chemokine 2 precursor
ENSMUSG00000025746	*Il6*	9.95	Interleukin-6 precursor
ENSMUSG00000018930	*Ccl4*	9.87	C-C motif chemokine 4 precursor
ENSMUSG00000000982	*Ccl3*	9.35	C-C motif chemokine 3 precursor
ENSMUSG00000034855	*Cxcl10*	9.03	C-X-C motif chemokine 10 precursor
ENSMUSG00000027398	*Il1b*	8.70	Interleukin-1 beta precursor
ENSMUSG00000029379	*Cxcl3*	8.68	C-X-C motif chemokine 3 precursor
ENSMUSG00000026166	*Ccl20*	8.65	C-C motif chemokine 20 Precursor
ENSMUSG00000029380	*Cxcl1*	8.61	C-X-C motif chemokine 1 precursor
ENSMUSG00000035385	*Ccl2*	8.49	C-C motif chemokine 2 precursor
ENSMUSG00000025383	*Il23α*	8.00	Interleukin-23 subunit alpha precursor
ENSMUSG00000027399	*Il1α*	7.92	Interleukin-1 alpha precursor
ENSMUSG00000016529	*Il10*	7.83	Interleukin-10 precursor
ENSMUSG00000060183	*Cxcl11*	7.41	C-X-C motif chemokine 11 precursor
ENSMUSG00000029417	*Cxcl9*	7.32	C-X-C motif chemokine 9 precursor
ENSMUSG00000044103	*Il36*	6.82	Interleukin-36 gamma
ENSMUSG00000035373	*Ccl7*	6.76	C-C motif chemokine 7 precursor
ENSMUSG00000024401	*Tnf*	6.49	Tumor necrosis factor isoform 1
ENSMUSG00000041872	*Il17f*	6.44	Interleukin-17F precursor
ENSMUSG00000020676	*Ccl11*	6.38	Eotaxin precursor
ENSMUSG00000025929	*IL-17a*	5.61	Interleukin-17A precursor
ENSMUSG00000029371	*Cxcl5*	5.39	C-X-C motif chemokine 5 precursor
ENSMUSG00000026984	*Il36*	4.93	Interleukin-36 alpha
ENSMUSG00000091938	*Ccl19*	4.93	C-C motif chemokine 19-like
ENSMUSG00000031780	*Ccl12*	4.93	C-C motif chemokine 12 Precursor
ENSMUSG00000074695	*Il22*	4.88	Interleukin-22 precursor
ENSMUSG00000055170	*Ifng*	4.76	Interferon gamma precursor
ENSMUSG00000019122	*Ccl9*	4.50	C-C motif chemokine 9 precursor
ENSMUSG00000044701	*Il27α*	4.15	Interleukin-27 subunit alpha precursor
ENSMUSG00000071005	*Ccl19*	3.91	C-C motif chemokine 19 precursor
ENSMUSG00000003206	*Il27*	3.91	Interleukin-27 subunit beta precursor

ENSMUSG00000004296	*Il12*	3.66	Interleukin-12 subunit beta precursor
ENSMUSG00000031779	*Ccl22*	3.48	C-C motif chemokine 22 precursor
ENSMUSG00000028435	*Ccl12*	3.27	C-C motif chemokine 12 precursor
ENSMUSG00000016524	*Il19*	3.25	Interleukin-19 precursor
ENSMUSG00000009185	*Ccl8*	3.17	C-C motif chemokine 8 precursor
ENSMUSG00000030748	*Il4α*	3.06	Interleukin-4 receptor subunit alpha precursor
ENSMUSG00000023078	*Cxcl13*	2.89	C-X-C motif chemokine 13 precursor
ENSMUSG00000031712	*Il15*	2.70	Interleukin 15 Precursor
ENSMUSG00000027776	*Il12a*	2.55	Interleukin-12 subunit alpha isoform 1
ENSMUSG00000040329	*Il7*	2.47	Interleukin-7 precursor
ENSMUSG00000001741	*Il6*	2.24	Pro-interleukin-16
ENSMUSG00000024810	*Il33*	2.19	Interleukin-33 precursor
