# Supplementary material for: Transcript profiling of the immunological interactions between Actinobacillus pleuropneumoniae serotype 7 and the host by dual RNA-seq
Source: BMC Microbiol. 2017 Sep 12;17:193. doi: 10.1186/s12866-017-1105-4 (PMC5596872; doi:10.1186/s12866-017-1105-4)
Supplement: Supplementary file 4 — Gene-specific primers for qRT-PCR. Significant DEGs of App and Mmu were selected to test the RNA-seq results. The DEGs of App included adhE, dmsA, dmsB, fruK, malE, lamB, hiuH, MetQ1, and the DEGs of Mmu included Cxcl2, Csf3, Ccl4, Ccl3, Tpm3, 60SrDNA, Csnk2a1, Hbα. 16SrDNA and GAPDH were used as the reference genes of App and Mmu, respectively. (PDF 182 kb) [file 12866_2017_1105_MOESM4_ESM.pdf]

Additional file 4: Gene-specific primers for qRT-PCR.

Gene Name	Primer sequences (5'-3')	Gene Name	Primer sequences (5'-3')
adhE	Adh-S: GGAACTCGCCATCAAC<br>Adh-A:TTCTCCGTCTGCCCACA	*Cxcl2*	Cxc-S: CACCAACCACCAGGCTAC<br>Cxc-A: GCAGGGTCTTCAGGCATT
dmsA	DmA-S: ATTCCTATTCGCCCTGG<br>DmA-A:CATCATCGCCTTGACCTA	*Csf3*	Csf3-S: GGAGTCTTGGTCCTACCGTTA<br>Csf3-A: GCTGGAAGGCAGAAGTGAA
dmsB	DmB-S:TGTGACGGTTGTTATTCTCG<br>DmB-A: TGCGTGTTTATTGGCTTT	*Ccl4*	Ccl4-S: TGCTACACGCTACGGAAC<br>Ccl4-A: GATGACCCTACATTAGACCC
fruK	Fru-S: AGCGGATGTGACAGACTTA<br>Fru-A: CCTTGTTGATGGAGCGAT	*Ccl3*	Ccl3-S: ATCTAAAGGGTGCCAGTG<br>Ccl3-A: GCATTCAGTTCCAGGTCA
malE	Mal-S: TTTCCCTCAAGTCGCCTC<br>Mal-A: TTTATTCGCATCCCAACC	*Tpm3*	Tpm-S: GCTGAACACCTTTGGGA<br>Tpm-A: GGCAGAAGTGCTTGGAAT
lamB	Lam-S: GGGAAAGTAGCCAAGATA<br>Lam-A: GTGCTCAATAGTTGCCATA	*60SrDNA*	60S-S: TCCTACTGGGTTGGTGAA<br>60S-A: TGTGGTGGAACTTGTGGC
hiuH	Hiu-S:CTTCCGTTTGTGCTGCTA<br>Hiu-A: CATCGGTAACTTCTTTCG	*Csnk2a1*	Csn-S: ATGTCTTACTAACCTAACCCTA<br>Csn-A: GCCCACTAACTAACTGTCTTC
metQ1	Met-S: TCCGACAAATCTTTATGC<br>Met-A:AATCTTTCGCTTCCACAA	*Hbα*	Hbα-A: CCAAGGCAGGGAACATA<br>Hbα-S: TGGAGGTCAGCACGGT
16SrDNA	16S-A: CCCTTATCCTTTGTTGCC<br>16S-S:CATCTTGCTTCCCTCTGTAT	*GAPDH*	Gap-A:CAGCAACTCCCACTCTTC<br>Gap-S:GTCCAGGGTTTCTTACTCC
